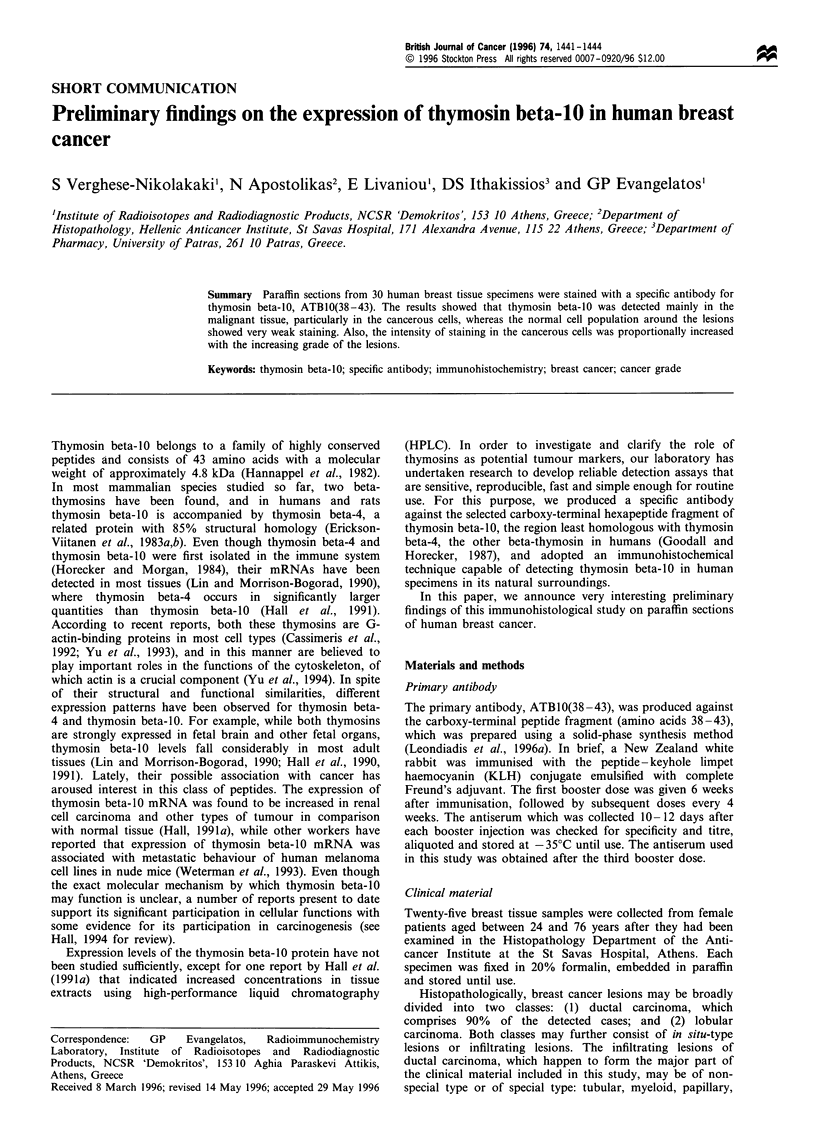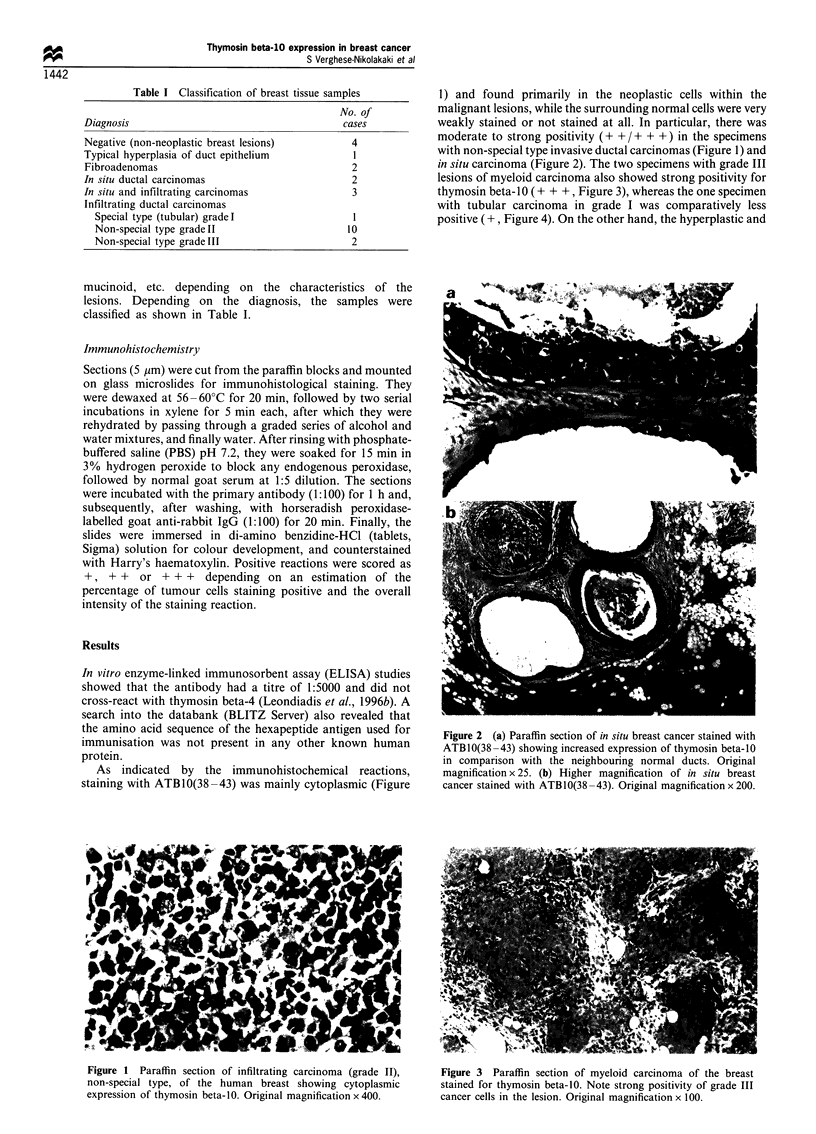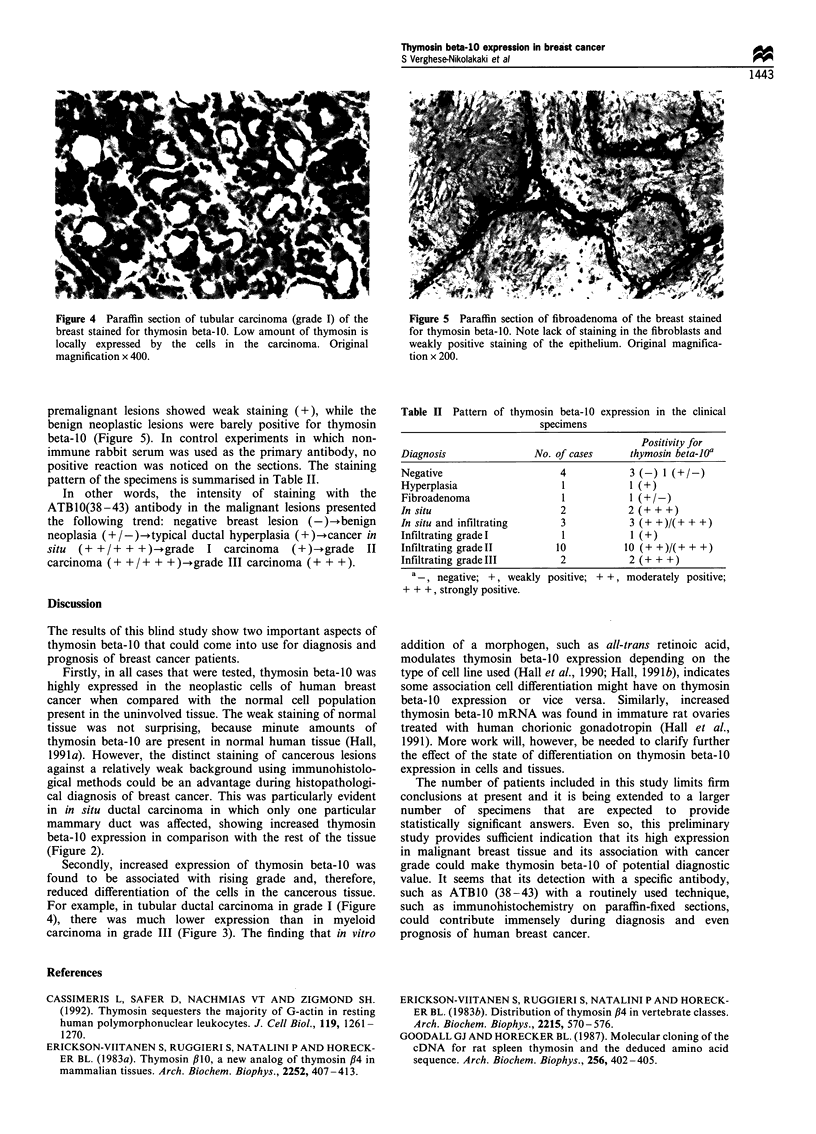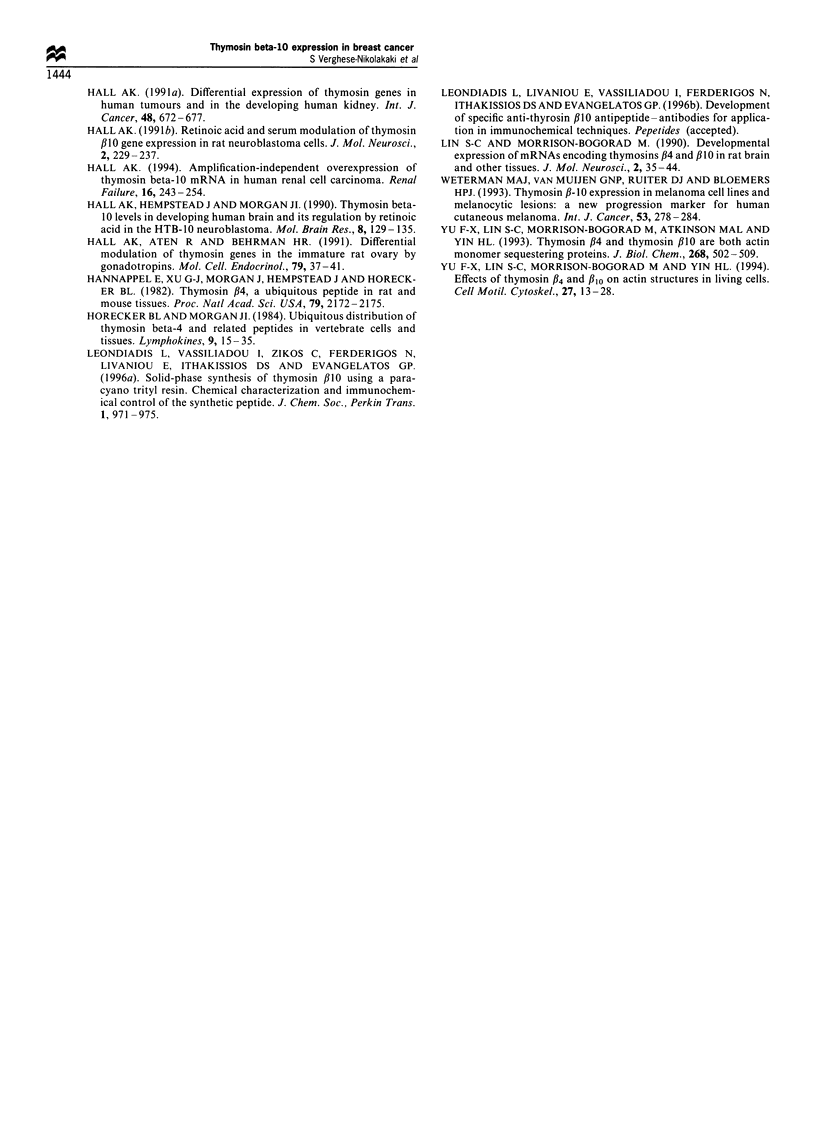# Preliminary findings on the expression of thymosin beta-10 in human breast cancer.

**DOI:** 10.1038/bjc.1996.562

**Published:** 1996-11

**Authors:** S. Verghese-Nikolakaki, N. Apostolikas, E. Livaniou, D. S. Ithakissios, G. P. Evangelatos

**Affiliations:** Institute of Radioisotopes and Radiodiagnostic Products, NCSR Demokritos, Athens, Greece.

## Abstract

**Images:**


British Journal of Cancer (1996) 74, 1441-1444
© 1996 Stockton Press All rights reserved 0007-0920/96 $12.00

SHORT COMMUNICATION

### Preliminary findings on the expression of thymosin beta-10 in human breast cancer

S Verghese-Nikolakaki[1], N Apostolikas[2], E Livaniou[1], DS Ithakissios[3] and GP Evangelatos[1]

[1]*Institute of Radioisotopes and Radiodiagnostic Products, NCSR 'Demokritos', 153 10 Athens, Greece;* [2]*Department of Histopathology, Hellenic Anticancer Institute, St Savas Hospital, 171 Alexandra Avenue, 115 22 Athens, Greece;* [3]*Department of Pharmacy, University of Patras, 261 10 Patras, Greece.*

**Summary** Paraffin sections from 30 human breast tissue specimens were stained with a specific antibody for thymosin beta-10, ATB10(38-43). The results showed that thymosin beta-10 was detected mainly in the malignant tissue, particularly in the cancerous cells, whereas the normal cell population around the lesions showed very weak staining. Also, the intensity of staining in the cancerous cells was proportionally increased with the increasing grade of the lesions.

**Keywords:** thymosin beta-10; specific antibody; immunohistochemistry; breast cancer; cancer grade

Thymosin beta-10 belongs to a family of highly conserved peptides and consists of 43 amino acids with a molecular weight of approximately 4.8 kDa (Hannappel *et al.*, 1982). In most mammalian species studied so far, two beta-thymosins have been found, and in humans and rats thymosin beta-10 is accompanied by thymosin beta-4, a related protein with 85% structural homology (Erickson-Viitanen *et al.*, 1983*a,b*). Even though thymosin beta-4 and thymosin beta-10 were first isolated in the immune system (Horecker and Morgan, 1984), their mRNAs have been detected in most tissues (Lin and Morrison-Bogorad, 1990), where thymosin beta-4 occurs in significantly larger quantities than thymosin beta-10 (Hall *et al.*, 1991). According to recent reports, both these thymosins are G-actin-binding proteins in most cell types (Cassimeris *et al.*, 1992; Yu *et al.*, 1993), and in this manner are believed to play important roles in the functions of the cytoskeleton, of which actin is a crucial component (Yu *et al.*, 1994). In spite of their structural and functional similarities, different expression patterns have been observed for thymosin beta-4 and thymosin beta-10. For example, while both thymosins are strongly expressed in fetal brain and other fetal organs, thymosin beta-10 levels fall considerably in most adult tissues (Lin and Morrison-Bogorad, 1990; Hall *et al.*, 1990, 1991). Lately, their possible association with cancer has aroused interest in this class of peptides. The expression of thymosin beta-10 mRNA was found to be increased in renal cell carcinoma and other types of tumour in comparison with normal tissue (Hall, 1991*a*), while other workers have reported that expression of thymosin beta-10 mRNA was associated with metastatic behaviour of human melanoma cell lines in nude mice (Weterman *et al.*, 1993). Even though the exact molecular mechanism by which thymosin beta-10 may function is unclear, a number of reports present to date support its significant participation in cellular functions with some evidence for its participation in carcinogenesis (see Hall, 1994 for review).

Expression levels of the thymosin beta-10 protein have not been studied sufficiently, except for one report by Hall *et al.* (1991*a*) that indicated increased concentrations in tissue extracts using high-performance liquid chromatography (HPLC). In order to investigate and clarify the role of thymosins as potential tumour markers, our laboratory has undertaken research to develop reliable detection assays that are sensitive, reproducible, fast and simple enough for routine use. For this purpose, we produced a specific antibody against the selected carboxy-terminal hexapeptide fragment of thymosin beta-10, the region least homologous with thymosin beta-4, the other beta-thymosin in humans (Goodall and Horecker, 1987), and adopted an immunohistochemical technique capable of detecting thymosin beta-10 in human specimens in its natural surroundings.

In this paper, we announce very interesting preliminary findings of this immunohistological study on paraffin sections of human breast cancer.

### Materials and methods

### *Primary antibody*

The primary antibody, ATB10(38-43), was produced against the carboxy-terminal peptide fragment (amino acids 38-43), which was prepared using a solid-phase synthesis method (Leondiadis *et al.*, 1996*a*). In brief, a New Zealand white rabbit was immunised with the peptide-keyhole limpet haemocyanin (KLH) conjugate emulsified with complete Freund's adjuvant. The first booster dose was given 6 weeks after immunisation, followed by subsequent doses every 4 weeks. The antiserum which was collected 10-12 days after each booster injection was checked for specificity and titre, aliquoted and stored at −35°C until use. The antiserum used in this study was obtained after the third booster dose.

### *Clinical material*

Twenty-five breast tissue samples were collected from female patients aged between 24 and 76 years after they had been examined in the Histopathology Department of the Anti-cancer Institute at the St Savas Hospital, Athens. Each specimen was fixed in 20% formalin, embedded in paraffin and stored until use.

Histopathologically, breast cancer lesions may be broadly divided into two classes: (1) ductal carcinoma, which comprises 90% of the detected cases; and (2) lobular carcinoma. Both classes may further consist of *in situ*-type lesions or infiltrating lesions. The infiltrating lesions of ductal carcinoma, which happen to form the major part of the clinical material included in this study, may be of non-special type or of special type: tubular, myeloid, papillary,

Correspondence: GP Evangelatos, Radioimmunochemistry Laboratory, Institute of Radioisotopes and Radiodiagnostic Products, NCSR 'Demokritos', 153 10 Aghia Paraskevi Attikis, Athens, Greece
Received 8 March 1996; revised 14 May 1996; accepted 29 May 1996

Table I Classification of breast tissue samples

| *Diagnosis* | *No. of cases* |
|---|---|
| Negative (non-neoplastic breast lesions) | 4 |
| Typical hyperplasia of duct epithelium | 1 |
| Fibroadenomas | 2 |
| *In situ* ductal carcinomas | 2 |
| *In situ* and infiltrating carcinomas | 3 |
| Infiltrating ductal carcinomas | |
| Special type (tubular) grade I | 1 |
| Non-special type grade II | 10 |
| Non-special type grade III | 2 |

mucinoid, etc. depending on the characteristics of the lesions. Depending on the diagnosis, the samples were classified as shown in Table I.

*Immunohistochemistry*

Sections (5 μm) were cut from the paraffin blocks and mounted on glass microslides for immunohistological staining. They were dewaxed at 56–60°C for 20 min, followed by two serial incubations in xylene for 5 min each, after which they were rehydrated by passing through a graded series of alcohol and water mixtures, and finally water. After rinsing with phosphate-buffered saline (PBS) pH 7.2, they were soaked for 15 min in 3% hydrogen peroxide to block any endogenous peroxidase, followed by normal goat serum at 1:5 dilution. The sections were incubated with the primary antibody (1:100) for 1 h and, subsequently, after washing, with horseradish peroxidase-labelled goat anti-rabbit IgG (1:100) for 20 min. Finally, the slides were immersed in di-amino benzidine-HCl (tablets, Sigma) solution for colour development, and counterstained with Harry's haematoxylin. Positive reactions were scored as +, ++ or +++ depending on an estimation of the percentage of tumour cells staining positive and the overall intensity of the staining reaction.

### Results

*In vitro* enzyme-linked immunosorbent assay (ELISA) studies showed that the antibody had a titre of 1:5000 and did not cross-react with thymosin beta-4 (Leondiadis *et al*., 1996*b*). A search into the databank (BLITZ Server) also revealed that the amino acid sequence of the hexapeptide antigen used for immunisation was not present in any other known human protein.

As indicated by the immunohistochemical reactions, staining with ATB10(38–43) was mainly cytoplasmic (Figure 1) and found primarily in the neoplastic cells within the malignant lesions, while the surrounding normal cells were very weakly stained or not stained at all. In particular, there was moderate to strong positivity (++/+++) in the specimens with non-special type invasive ductal carcinomas (Figure 1) and *in situ* carcinoma (Figure 2). The two specimens with grade III lesions of myeloid carcinoma also showed strong positivity for thymosin beta-10 (+++, Figure 3), whereas the one specimen with tubular carcinoma in grade I was comparatively less positive (+, Figure 4). On the other hand, the hyperplastic and

**Figure 2** (**a**) Paraffin section of *in situ* breast cancer stained with ATB10(38–43) showing increased expression of thymosin beta-10 in comparison with the neighbouring normal ducts. Original magnification ×25. (**b**) Higher magnification of *in situ* breast cancer stained with ATB10(38–43). Original magnification ×200.

**Figure 1** Paraffin section of infiltrating carcinoma (grade II), non-special type, of the human breast showing cytoplasmic expression of thymosin beta-10. Original magnification ×400.

**Figure 3** Paraffin section of myeloid carcinoma of the breast stained for thymosin beta-10. Note strong positivity of grade III cancer cells in the lesion. Original magnification ×100.

**Figure 4** Paraffin section of tubular carcinoma (grade I) of the breast stained for thymosin beta-10. Low amount of thymosin is locally expressed by the cells in the carcinoma. Original magnification x 400.

**Figure 5** Paraffin section of fibroadenoma of the breast stained for thymosin beta-10. Note lack of staining in the fibroblasts and weakly positive staining of the epithelium. Original magnification x 200.

premalignant lesions showed weak staining (+), while the benign neoplastic lesions were barely positive for thymosin beta-10 (Figure 5). In control experiments in which non-immune rabbit serum was used as the primary antibody, no positive reaction was noticed on the sections. The staining pattern of the specimens is summarised in Table II.

In other words, the intensity of staining with the ATB10(38–43) antibody in the malignant lesions presented the following trend: negative breast lesion (−)→benign neoplasia (+/−)→typical ductal hyperplasia (+)→cancer *in situ* (++/+++)→grade I carcinoma (+)→grade II carcinoma (++/+++)→grade III carcinoma (+++).

**Table II** Pattern of thymosin beta-10 expression in the clinical specimens

| *Diagnosis* | *No. of cases* | *Positivity for thymosin beta-10*[a] |
|---|---|---|
| Negative | 4 | 3 (−) 1 (+/−) |
| Hyperplasia | 1 | 1 (+) |
| Fibroadenoma | 1 | 1 (+/−) |
| *In situ* | 2 | 2 (+++) |
| *In situ* and infiltrating | 3 | 3 (++)/(+++) |
| Infiltrating grade I | 1 | 1 (+) |
| Infiltrating grade II | 10 | 10 (++)/(+++) |
| Infiltrating grade III | 2 | 2 (+++) |

[a]−, negative; +, weakly positive; ++, moderately positive; +++, strongly positive.

### Discussion

The results of this blind study show two important aspects of thymosin beta-10 that could come into use for diagnosis and prognosis of breast cancer patients.

Firstly, in all cases that were tested, thymosin beta-10 was highly expressed in the neoplastic cells of human breast cancer when compared with the normal cell population present in the uninvolved tissue. The weak staining of normal tissue was not surprising, because minute amounts of thymosin beta-10 are present in normal human tissue (Hall, 1991*a*). However, the distinct staining of cancerous lesions against a relatively weak background using immunohistological methods could be an advantage during histopathological diagnosis of breast cancer. This was particularly evident in *in situ* ductal carcinoma in which only one particular mammary duct was affected, showing increased thymosin beta-10 expression in comparison with the rest of the tissue (Figure 2).

Secondly, increased expression of thymosin beta-10 was found to be associated with rising grade and, therefore, reduced differentiation of the cells in the cancerous tissue. For example, in tubular ductal carcinoma in grade I (Figure 4), there was much lower expression than in myeloid carcinoma in grade III (Figure 3). The finding that *in vitro* addition of a morphogen, such as *all-trans* retinoic acid, modulates thymosin beta-10 expression depending on the type of cell line used (Hall *et al*., 1990; Hall, 1991*b*), indicates some association cell differentiation might have on thymosin beta-10 expression or vice versa. Similarly, increased thymosin beta-10 mRNA was found in immature rat ovaries treated with human chorionic gonadotropin (Hall *et al*., 1991). More work will, however, be needed to clarify further the effect of the state of differentiation on thymosin beta-10 expression in cells and tissues.

The number of patients included in this study limits firm conclusions at present and it is being extended to a larger number of specimens that are expected to provide statistically significant answers. Even so, this preliminary study provides sufficient indication that its high expression in malignant breast tissue and its association with cancer grade could make thymosin beta-10 of potential diagnostic value. It seems that its detection with a specific antibody, such as ATB10 (38–43) with a routinely used technique, such as immunohistochemistry on paraffin-fixed sections, could contribute immensely during diagnosis and even prognosis of human breast cancer.

### References

CASSIMERIS L, SAFER D, NACHMIAS VT AND ZIGMOND SH. (1992). Thymosin sequesters the majority of G-actin in resting human polymorphonuclear leukocytes. *J. Cell Biol*., **119**, 1261–1270.

ERICKSON-VIITANEN S, RUGGIERI S, NATALINI P AND HORECKER BL. (1983*a*). Thymosin $\beta$10, a new analog of thymosin $\beta$4 in mammalian tissues. *Arch. Biochem. Biophys*., **2252**, 407–413.

ERICKSON-VIITANEN S, RUGGIERI S, NATALINI P AND HORECKER BL. (1983*b*). Distribution of thymosin $\beta$4 in vertebrate classes. *Arch. Biochem. Biophys*., **2215**, 570–576.

GOODALL GJ AND HORECKER BL. (1987). Molecular cloning of the cDNA for rat spleen thymosin and the deduced amino acid sequence. *Arch. Biochem. Biophys*., **256**, 402–405.

HALL AK. (1991*a*). Differential expression of thymosin genes in human tumours and in the developing human kidney. *Int. J. Cancer*, **48,** 672–677.

HALL AK. (1991*b*). Retinoic acid and serum modulation of thymosin $\beta$10 gene expression in rat neuroblastoma cells. *J. Mol. Neurosci.*, **2,** 229–237.

HALL AK. (1994). Amplification-independent overexpression of thymosin beta-10 mRNA in human renal cell carcinoma. *Renal Failure*, **16,** 243–254.

HALL AK, HEMPSTEAD J AND MORGAN JI. (1990). Thymosin beta-10 levels in developing human brain and its regulation by retinoic acid in the HTB-10 neuroblastoma. *Mol. Brain Res.*, **8,** 129–135.

HALL AK, ATEN R AND BEHRMAN HR. (1991). Differential modulation of thymosin genes in the immature rat ovary by gonadotropins. *Mol. Cell. Endocrinol.*, **79,** 37–41.

HANNAPPEL E, XU G-J, MORGAN J, HEMPSTEAD J AND HORECKER BL. (1982). Thymosin $\beta$4, a ubiquitous peptide in rat and mouse tissues. *Proc. Natl Acad. Sci. USA*, **79,** 2172–2175.

HORECKER BL AND MORGAN JI. (1984). Ubiquitous distribution of thymosin beta-4 and related peptides in vertebrate cells and tissues. *Lymphokines*, **9,** 15–35.

LEONDIADIS L, VASSILIADOU I, ZIKOS C, FERDERIGOS N, LIVANIOU E, ITHAKISSIOS DS AND EVANGELATOS GP. (1996*a*). Solid-phase synthesis of thymosin $\beta$10 using a para-cyano trityl resin. Chemical characterization and immunochemical control of the synthetic peptide. *J. Chem. Soc., Perkin Trans.* **1,** 971–975.

LEONDIADIS L, LIVANIOU E, VASSILIADOU I, FERDERIGOS N, ITHAKISSIOS DS AND EVANGELATOS GP. (1996b). Development of specific anti-thyrosin $\beta$10 antipeptide–antibodies for application in immunochemical techniques. *Pepetides* (accepted).

LIN S-C AND MORRISON-BOGORAD M. (1990). Developmental expression of mRNAs encoding thymosins $\beta$4 and $\beta$10 in rat brain and other tissues. *J. Mol. Neurosci.*, **2,** 35–44.

WETERMAN MAJ, VAN MUIJEN GNP, RUITER DJ AND BLOEMERS HPJ. (1993). Thymosin $\beta$-10 expression in melanoma cell lines and melanocytic lesions: a new progression marker for human cutaneous melanoma. *Int. J. Cancer*, **53,** 278–284.

YU F-X, LIN S-C, MORRISON-BOGORAD M, ATKINSON MAL AND YIN HL. (1993). Thymosin $\beta$4 and thymosin $\beta$10 are both actin monomer sequestering proteins. *J. Biol. Chem.*, **268,** 502–509.

YU F-X, LIN S-C, MORRISON-BOGORAD M AND YIN HL. (1994). Effects of thymosin $\beta_4$ and $\beta_{10}$ on actin structures in living cells. *Cell Motil. Cytoskel.*, **27,** 13–28.